# Temporal‐Parotid Resection for Malignant Parotid Tumors: A Systematic Review

**DOI:** 10.1002/lary.32267

**Published:** 2025-05-14

**Authors:** Antonio Daloiso, Diego Cazzador, Giulia Tealdo, Gioia Martini, Leonardo Franz, Antonio Mazzoni, Piero Nicolai, Elisabetta Zanoletti

**Affiliations:** ^1^ Section of Otorhinolaryngology – Head and Neck Surgery Department of Neuroscience, University of Padova Padova Italy; ^2^ Unit of Otorhinolaryngology – Head and Neck Surgery Padua University Hospital Padova Italy; ^3^ Unit of Phoniatrics and Audiology, Ospedale Ca' Foncello Treviso ‐ ULSS 2 Marca Trevigiana, Department of Neurosciences University of Padova Treviso Italy

**Keywords:** facial nerve, head and neck cancer, parotid cancer, survival, systematic review, temporal bone resection, temporal‐parotid resection

## Abstract

**Objective:**

Surgery involving the skull base has recently gained significant prominence in treatment of head and neck cancer. “En bloc temporal‐parotid resection,” as applied in primary malignancies of the external ear, should be considered as a shift in the surgical landscape of “extended total parotidectomies” to achieve negative surgical margins and decrease the risk of tumor spillage during the procedure. The purpose of this systematic review is to evaluate survival outcomes and margin status in patients with parotid malignancies involving the temporal bone and treated with temporal‐parotid resection (TPR).

**Data Sources:**

PubMed, Scopus, and Embase databases were systematically searched until 11 February 2025.

**Review Methods:**

The protocol of this investigation was registered on PROSPERO in February 2024 and the systematic review was performed according to the PRISMA method.

**Results:**

The study included 336 patients from 13 articles published between 1993 and 2019. Negative surgical margins were achieved in 74.5% of cases. After a median follow‐up of 32 months, 64.0% of patients achieved local disease control. Most patients (51.4%) experienced distant metastases during follow‐up. Three‐year overall survival (OS) and disease‐specific survival (DSS) ranged between 72.4%–57.1% and 79.0%–66.7%, respectively.

**Conclusion:**

TPR represents a viable strategy to achieve safe oncological margins and adequate local disease control in patients with parotid gland malignancies infiltrating or abutting the temporal bone. Our findings support the growing interest in TPR, highlighting the need for further studies to refine patient selection criteria, surgical techniques, and postoperative management strategies.

**Trial Registration:** PROSPERO: CRD42024512013

**Level of Evidence:**

NA.

## Graphical abstract

Parotid gland tumors represent a complex surgical challenge. Temporal‐parotid resection (TPR) is indicated to manage temporal bone invasion. Thirteen studies investigated the role of TPR in literature, including 336 patients. High rates of negative margins and local disease control are achieved with TPR.

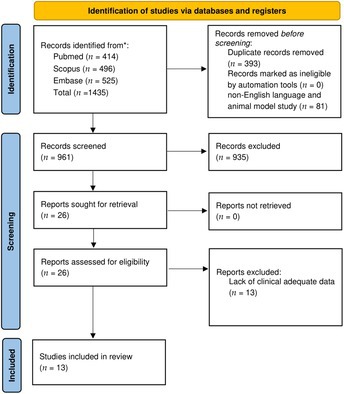

## 1 Introduction

Parotid gland tumors comprise 5% of head and neck cancers in Europe. The worldwide crude and age‐adjusted incidence of salivary gland cancer rates are 0.28 cases per 100,000 people per year, with 55,083 new patients diagnosed in 2022 [1]. Advanced high‐grade cases carry a poor prognosis; while the rate of overall survival at 2 years is 80% [2], a significant decline to 35% is observed at 5 years [3]. Surgery remains the cornerstone of treatment for parotid malignancies [3, 4], although in cases of advanced tumors with extraglandular spread extending to adjacent tissues such as the mandible, external auditory canal, temporomandibular joint, and infratemporal fossa, the traditional enlarged or extended parotidectomy techniques may fail to achieve complete resection [3, 5]. Positive margins are recognized as a primary cause of treatment failure and recurrence [4, 6, 7, 8]. When tumors abut the mastoid or tympanic bone periosteum, it can be difficult to provide negative margins if an adequate portion of the temporal bone is not included in the specimen. A cervical‐parotid approach is associated with the risk of carrying the dissection in close proximity to the tumor or, even worse, violating it [2]. Furthermore, the periosteum and temporal bone are not effective barriers to tumor spread, but rather pathways for macroscopic and microscopic tumor dissemination [9, 10]. In this setting, extended or enlarged traditional parotidectomies risk being ineffective.

When large tumors involve or closely abut to the stylomastoid foramen, making identification of the facial nerve trunk difficult, the traditional dissection techniques in this narrow space involve a risk of both margin violation and tumor spillage [4, 6, 11]. To overcome these limitations, the concept of “en bloc temporal‐parotid resection (TPR)” was proposed as an alternative to traditional “enlarged parotidectomies” [9], with the intent of improving survival outcomes in patients with tumors invading the temporal bone. TPR is a resection of a variable portion of the temporal bone en bloc with the parotid and adjacent structures. The procedure is similar to the en bloc resection performed in primary temporal bone carcinoma [12, 13], which was described for the first time in the 1960s by Allen [14] and sporadically reappraised in subsequent years [4, 15, 16]. The analysis of the literature reflects the paucity of studies on TPR and the lack of pooled data in systematic reviews.

The aim of this review was to explore and summarize the current literature on surgical management with en bloc TPR for parotid malignancies involving the temporal bone, under the hypothesis that TPR can be a viable strategy to achieve negative surgical margins and improve local disease control in malignant parotid tumors involving or abutting the temporal bone.

## 2 Materials and Methods

### 2.1 Protocol Registration

Prior to study commencement, the protocol of this systematic review was registered in the International Prospective Register of Systematic Reviews (PROSPERO).

### 2.2 Search Strategy

A systematic literature review was conducted according to the Preferred Reporting Items for Systematic Reviews and Meta‐Analyses (PRISMA) recommendations [17]. The electronic databases Scopus, PubMed, and Embase were searched from database inception to 11 February 2025.

The inclusion criteria for selection of studies were chosen according to the PICOS tool: Patients (P), adults affected by malignant parotid tumors involving or abutting the temporal bone; Intervention (I), TPR; Comparator (C), none; Outcomes (O), Achievement of negative surgical margins, local disease control, and survival outcomes; Study design (S), retrospective and prospective cohort studies.

A combination of MeSH terms and free‐text words was utilized (see Supporting Information A). The reference lists of all the articles included were thoroughly screened to find other relevant publications. References were exported to Zotero bibliography manager (v6.0.10, Center for History and New Media, George Mason University, Fairfax, VA, USA). After removal of duplicates, two reviewers (D.C. and G.T.) independently screened all titles and abstracts and then evaluated the full texts of the eligible articles based on inclusion criteria. Any disagreement between the reviewers involved in the literature search was resolved through discussion with all authors to reach a consensus.

### 2.3 Selection Criteria

Studies were deemed eligible when the following inclusion criteria were met: (i) confirmed histopathological diagnosis of primary or secondary parotid malignancy; (ii) patients treated with TPR for the above‐mentioned tumors involving or abutting the temporal bone; (iii) studies reporting at least five cases. Exclusion criteria were as follows: (i) lack of relevant data (sex, age, number of patients, oncological status and features, essential surgical details); (ii) nonoriginal studies (i.e., reviews, recommendations, editorials, conference paper, clinical challenge, and book chapters); (iii) animal model studies; (iv) non‐English studies.

### 2.4 Data Extraction and Quality Assessment

Extracted data were collected in an electronic database, including first author, year of publication, sample size, patient age, sex, surgical approach, facial nerve assessment, reconstructive method, and outcomes. The quality of the studies eligible for inclusion was categorized as Poor, Fair, and Good, in agreement with the National Institutes of Health quality assessment tool for Observational Cohorts and Cross‐Sectional Studies (https://www.nhlbi.nih.gov/health‐topics/study‐quality‐assessment‐tools, accessed on 23 February 2024) [18]. Two reviewers (A.D. and D.C.) independently evaluated the papers, and any disagreement was resolved by discussion.

Risk of bias assessment for non‐randomized studies was performed with the Risk of Bias in Non‐randomized Studies of Interventions (ROBINS‐I) tool [19]. Importantly, ROBINS‐I bias assessments are made based on the comparison between a given study and a theoretical randomized‐controlled trial with ideal design for the study question, and the latter represents the standard for a “low‐risk” study.

## 3 Results

### 3.1 Search Results, Quality Assessment and Risk of Bias

**FIGURE 1:**
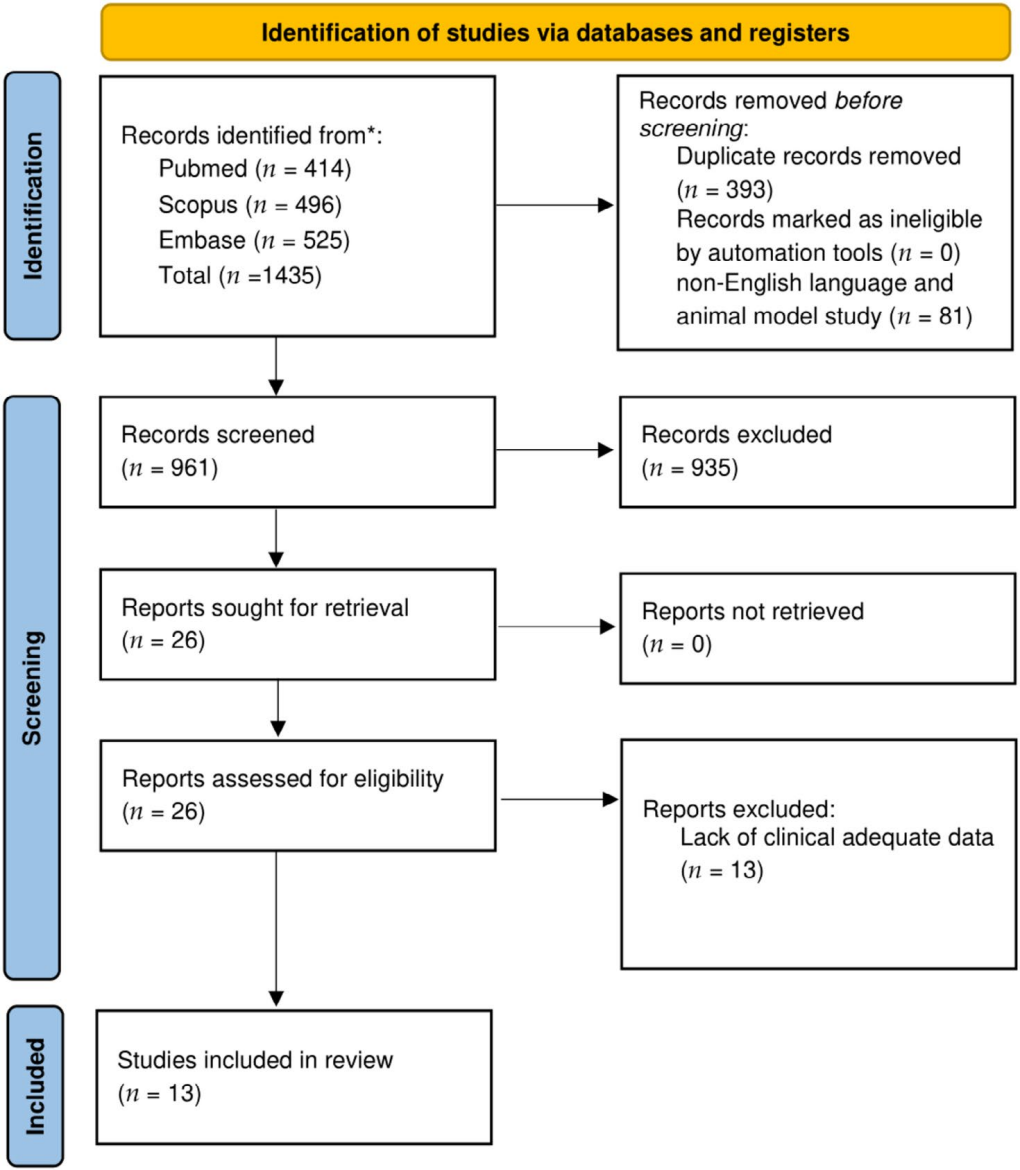
PRISMA diagram showing electronic database search and inclusion/exclusion process of the review. *Date of last search 11 February 2025. [Color figure can be viewed in the online issue, which is available at www.laryngoscope.com.]

A total of 1435 titles were collected from the literature search. After removal of duplicates and exclusion of 935 records that did not meet inclusion/exclusion criteria, 29 articles relevant to the topic were examined. No records were available for retrieving. Finally, 13 were included in the review [2, 4, 5, 6, 7, 11, 15, 16, 20, 21, 22, 23, 24]. A detailed flowchart of the search process is shown in Figure 1.

In accordance with the National Institutes of Health quality assessment tool for Observational Cohorts and Cross‐Sectional Studies [18], 4 studies (30.8%) were deemed of Good quality, 6 (46.2%) Fair, and 3 (23%) Poor, since they did not report clinical data (see Table A).

Risk of bias summaries for nonrandomized studies are given in Figure S1. No study was assessed as “critical risk,” and eight were scored as “serious risk” primarily due to confounding and missing data in the results. Finally, five studies were classified as “moderate risk.”

### 3.2 Characteristics of Studies Included

All studies included were retrospective case‐series published between 1993 and 2019, encompassing patients treated from 1987 to 2016 (Table 1) [2, 4, 5, 6, 7, 11, 15, 16, 20, 21, 22, 23, 24]. Most of the articles were from the United States (61.5%). The median number of patients per study was 16 (range 7–120).

**TABLE 1:** Characteristics of the studies included in the systematic review.

Author	Period	Country	Patients (*n*)	Mean age (year)	Sex (M/F)
Amin et al. 2017 [5]	2006–2011	Egypt	8	56.1 (40–69)	3/5
Aslier et al. 2019 [24]	1995–2016	Turkey	7	55.6 (32–75)	4/3
Carlson et al. 2015 [7]^a^	1994–2014	USA	30	58.4 (34–80)	23/7
Gidley et al. 2011 [6]	2002–2009	USA	49	NA (14.3–90.9)	33/16
Kadakia et al. 2017 [16]	1998–2014	USA	120	67.6 (19–87)	82/38
Leonetti et al. 1993 [21]	1987–1991	USA	27	59.4 (27–75)	15/12
Leonetti et al. 2008 [20]	1988–2004	USA	16	46.8 (14–62)	7/9
Martin et al. 2017 [15]	2007–2013	USA	15	63 (39–85)	9/6
Mehra et al. 2011 [2]	1994–2010	USA	12	62 (33–88)	8/4
Munir et al. 2012 [4]	2008–2010	UK	8	73.9 (63–88)	6/2
Sargi et al. 2010 [22]^a^	1999–2008	USA	17	NA	NA
Wierzbicka et al. 2016 [23]	2006–2010	Poland	9	51.2 (31–68)	3/6
Yokoyama et al. 2016 [11]	NA	Japan	18	59.3 (30–84)	9/9

Abbreviations: F = female; M = male; NA = not available.

^a^ Raw data not extractable.

The articles are discussed in the following paragraphs. Due to the high heterogeneity of the collected data, a meta‐analysis could not be performed.

### 3.3 Characteristics of Patients and Tumors

A total of 336 patients diagnosed with primary or secondary parotid malignancy and treated with TPR were included in the analysis. Sex was not available for 17 patients, and among the remaining there were 202 males (63.3%) and 117 females (36.7%). The mean age was 50.2 years (range: 14–90.9), while in two studies this information was not reported [6, 22]. Data on patient demographics are summarized in Table 1.

Table 2 details the clinical and radiological features of patients. Two hundred fifty patients (74.4%) presented with primary cancer, while 86 (25.5%) had a secondary lesion from skin or other cancer sites. Preoperative facial nerve status was reported in 9 studies [2, 4, 5, 6, 7, 15, 21, 23, 24]. In all, 100 of 165 patients had signs of facial nerve paralysis (60.6%) prior to surgery. House‐Brackmann grading was seldom reported.

**TABLE 2:** Characteristics of tumors in studies included in the systematic review.

Author	Tumor origin (primary vs. metastatic)	*T* (T1/T2/T3/T4a/T4b)	*N* (N0/N+)	*M* (M0/M1)	Histopathology of primary parotid tumor	Clinical and radiological features/invasion	Preoperative facial nerve assessment (HB scale)
Amin et al. 2017 [5]	8–0	1/2/0/4/1	8/0	NA	NA	HL 4 EAC 4; IJV 1; ICA 2 Intracranial 1	4/8 (V)
Aslier et al. 2019 [24]	7–0	0/0/0/7	4/3	7/0	ACC 1; SCC 2; Adenosquamous 1; Myoep 1; ADC 1; Spindle cell 1	EGS 7; Skin invasion 4 EAC 3; TMJ 2; ITF 5	4/7 (1‐V; 3‐VI)
Carlson et al. 2015 [7]^a^	22–8	0/0/0/20/10	18/12	30/0	NA	NA	23/30
Gidley et al. 2011 [6]	49–0	NA	NA	NA	MEC 1; ACC 5; SCC 14; AcCC 8; ADC 4; ex‐pleo 1; SDC 6; Other 10	HL 10; Otalgia 10; Trisma 6; EAC 19; Dura 2 Middle ear 2 Stylomastoid foramen 15	32/49
Kadakia et al. 2017 [16]	42–78	NA	NA	NA	MEC 20; ACC 6; AcCC 2; ex‐pleo 5; melanoma 2; myoep 3; sarcoma 4	NA	NA
Leonetti et al. 1993 [21]	27–0	NA	NA	NA	ADC 7; AcCC 3; MEC 3; ACC 4; SCC 3; ex‐pleo 2	Dysphagia 9; Dysphonia 5 HL 5; Trismus 2; Headache 2	8/27
Leonetti et al. 2008 [20]	16–0	NA	NA	NA	ACC 16	HL 7; Trismus 7; TMJ 9 EAC 7 Stylomastoid foramen 4	NA
Martin et al. 2017 [15]	15–0	0/0/2/12/1	12/3	NA	MEC 1; ACC 2; SCC 3; AcCC 1; ADC 2; ex‐pleo 3; SDC 1; Other 2	EGS 13 Stylomastoid foramen 12 Dura 1	12/15
Mehra et al. 2011 [2]	12–0	0/2/0/5/5	0/1	11/1	MEC 3; ACC 2; SCC 2; ex‐pleo 1; SDC 1; Other 3	NA	6/12
Munir et al. 2012 [4]	8–0	NA	NA	NA	ACC 2; SCC 3; ex‐pleo 1; ADC 1; Other 1	NA	5/8
Sargi et al. 2010 [22]^a^	17–0	NA	NA	NA	MEC 10; ACC 3; SCC 2; ex‐pleo 1; Undifferentiated 1	NA	NA
Wierzbicka et al. 2016 [23]	9–0	0/0/0/9	5/4	8/1	MEC 1; ACC 2; SCC 2; AcCC 1; ADC 1; Myoep 1; Undifferentiated 1	Skin invasion 4 EAC 9; TMJ 7; Dura 3 Middle ear 4; Inner ear 2	6/9 (2‐IV; 4‐VI)
Yokoyama et al. 2016 [11]	18–0	NA	NA	NA	AcCC 2; ADC 2; SCC 2; SDC 3; ex‐pleo 4; Other 5	NA	NA

Abbreviations: ACC = adenoid cystic carcinoma; AcCC = acinic cell carcinoma; ADC = adenocarcinoma; HB = House‐Brackmann; ex‐pleo = carcinoma ex‐pleomorphic adenoma; EAC = external auditory canal; EGS = extraglandular spread; IJV = internal jugular vein; ITF = infratemporal fossa; LTRB = lateral temporal bone resection; MEC = mucoepidermoid carcinoma; Myoep = myoepithelial carcinoma; NA = not available; SCC = squamous cell carcinoma; SDC = salivary duct carcinoma; TMJ = temporomandibular joint; TTBR = total temporal bone resection.

^a^ Raw data not extractable.

### 3.4 Treatment Strategies

Surgery included superficial, total, or radical parotidectomy combined with temporal bone resections variably extending to adjacent structures (Table 3). In detail, of 198 patients for whom the type of parotid resection was reported, 5 underwent superficial parotidectomy (2.5%), 63 total parotidectomy with facial nerve sparing (31.8%), and 130 radical parotidectomy with facial nerve sacrifice (65.7%). The extent of temporal bone resection varied from simple external auditory canal resection (5 cases, 1.8%), mastoidectomy (108, 38%), lateral temporal bone resection (129, 45.4%), and subtotal temporal bone resection (41, 14.4%). Total temporal bone resection was performed in one case (0.4%). Neck dissection was performed in 119 of 145 patients (82%). Regarding adjuvant treatments, 104 patients received radiotherapy, 4 received chemotherapy alone, and 20 received adjuvant chemo‐radiotherapy.

**TABLE 3:** Treatments of the studies included in the systematic review.

Author	Type of parotidectomy (partial‐total‐radical)	Type of temporal bone resection	Additional surgical treatment	Surgical margins	Surgical defect reconstruction	Facial nerve reconstruction	Adjuvant treatment
Amin et al. 2017 [5]	0–6–2	LTBR 6 STBR 2	ND 8 TMJ 2 Mand 2	Pos 2 Neg 6	RFFF 1; TMF 3 PMMF 3; LDMF 1	NA	RT 4
Aslier et al. 2019 [24]	0–0–7	LTBR 7	ND 5 Mand 2	Pos 2 Neg 5	POSRF 2; PFF 1 PMmyoF 2 PMMF 2	NA	CRT 5 CT 1
Carlson et al. 2015 [7]^a^	0–30–0	M 27 LTBR 3	ND 27 ITF 6	Pos 10 Neg 20	NA	17	RT 18 CRT 7
Gidley et al. 2011 [6]	5–8–35	M 33 LTBR 15 STBR 1	ND 45 Mand 8 ITF 6	Pos 6 Close 4 Neg 36 NA 3	ALT 18; RFFF 1 TMF 3; RAFF 3 SCM 3; CFF 2 PMMF 3; LDMF 1	21	RT 38 CT 3
Kadakia et al. 2017 [16]	NA	M 60 LTBR 60	NA	NA	NA	NA	NA
Leonetti et al. 1993 [21]	0–10–17	STBR 27	NA	NA	TMF 4 PMMF 3; LDMF 2 TRF 1; RAFF 8 LDMF + TRF 2	2	NA
Leonetti et al. 2008 [20]	0–0–16	EAC resection 5 LTBR 5 STBR 6	ND 4	Pos and close 4 Neg 12	NA	16 (Sural 2; RAFF 4; Serratus 10)	RT 16
Martin et al. 2017 [15]	0–1–14	LTBR 15	ND 15 Mand 2 Dural resection 1	Pos 6 Neg 9	ALT 11	NA	RT 5 CRT 7
Mehra et al. 2011 [2]	0–0–12	LTBR 12	ND 8	Pos 7 Neg 5	NA	NA	RT 9 CRT 2
Munir et al. 2012 [4]	0–1–7	M 8	ND 7	Neg 8	NA	7	RT 7
Sargi et al. 2010 [22]^a^	NA	NA	NA	NA	NA	NA	NA
Wierzbicka et al. 2016 [23]	0–0–9	LTBR 3 STBR 5 TTBR 1	NA	Pos 2 Neg 7	ALT 2 RFFF 4	2	RT 7
Yokoyama et al. 2016 [11]	0–7–11	M 15 LTBR 3	NA	Neg 18	NA	11	NA

Abbreviations: ALT = anterolateral thigh flap; CFF = cervico‐facial flap; CRT = chemoradiotherapy; CT = chemotherapy; EAC = external auditory canal; FF = free flap; FN = facial nerve; ITF = infratemporal fossa; LDMF = Latissimus dorsi myocutaneous flap; LTRB = lateral temporal bone resection; M = mastoidectomy; Mand = mandibulectomy; NA = not available; ND = neck dissection; Neg = negative margins; PFF = Parascapular free flap; PMMF = Pectoralis major myocutaneous flap; PMmyoF = Pectoralis major myofascial flap; pos = positive margins; POSRF = Parieto‐occipital scalp rotational flap; RAFF = Rectus abdominus free flap; RT = radiotherapy; STBR = subtotal temporal bone resection; TMF = Temporalis myocutaneous flap; TMJ = temporomandibular joint; TRF = Trapezius rotational flap; TTBR = total temporal bone resection.

^a^ Raw data not extractable.

Reconstruction of the surgical defect was performed with different techniques, ranging from primary closure to primary closure with fat graft obliteration, regional sliding or rotational flaps, and free flaps, as reported in Table 3. Facial nerve reconstruction and/or reinnervation were documented in 7 studies [4, 6, 7, 11, 20, 21, 23], in 81 of 157 (51.6%) patients.

### 3.5 Pathological Findings

Histopathological findings were reported in all but one study [5], while in one report the authors did not distinguish between primary and metastatic lesions [7]. In 220 patients with data available, the most frequent histologies were adenoid cystic carcinoma (*n* = 43, 19.5%), mucoepidermoid carcinoma (*n* = 39, 17.7%), squamous cell carcinoma (*n* = 33, 15%), adenocarcinoma (*n* = 18, 8.2%), and acinic cell carcinoma (*n* = 17, 7.7%). Other histological findings are reported in Table 2.

The status of surgical margins was reported in 169 patients. In 126 patients (74.5%) a R0 resection was reached, while in 43 cases the margins were positive or close (25.5%).

### 3.6 Survival

The median follow‐up period, when reported, was 32 months (range 0.1–208). In 291 patients with follow‐up information, 111 (38.1%) developed a relapse of disease. Among these, 40 (36.0%) experienced a local relapse, 14 (12.6%) lymph node metastasis, and 57 (51.4%) distant spread. The survival outcomes reported were overall survival (OS), disease‐specific survival (DSS), and disease‐free survival (DFS). Three‐year OS and DSS ranged between 57.1% and 72.4%, and between 66.7% and 79.0%, respectively. Other survival outcomes are summarized in Table 4.

**TABLE 4:** Outcomes of the included studies.

Author	Follow‐up, months (range)	Recurrence (L‐R‐D)	Time of recurrence, months (range)	Status (DOD‐DOC‐NED)	Overall survival	Other survival outcomes
Amin et al. 2017 [5]	18.3^a^ 18^b^ (10–22)	3–0–0	NA	2–0–6	43.8% (2 years)^d^	DSS 43.8% (2 years)^d^
Aslier et al. 2019 [24]	25.9^a^ 32^b^ (1–48)	0–0–4	NA	4–1–2	57.1% (3 years)^d^	DSS 66.7% (3 years)^d^
Carlson et al. 2015 [7]^c^	49.2^a^ 30.9^b^ (0–208)	4–4–8	26^a^ 19^b^	7–NA–NA	NA	DSS 83% (1 years); 79% (3 years); 72% (5 years)
Gidley et al. 2011 [6]	24.9^b^ (2.9–66.2)	8–3–7	7.6^a^ (1–25)	NA	72.4% (3 years)	NA
Kadakia et al. 2017 [16]	NA (18–132)	M group 13–0–7 LTBR group 2–0–9	NA	NA	NA	NA
Leonetti et al. 1993 [21]	31.8^a^ (12–60)	NA	NA	5–2–16	NA	NA
Leonetti et al. 2008 [20]	8.9y^a^ (2y–16 years)	3–0–3 (5 years)	NA	4–0–12 (5 years)	NA	NA
Martin et al. 2017 [15]	34.8^a^ (> 24)	2–2–6	11^a^	6–NA–NA	60% (2 years); 44% (5 years)	DFS 40% (2 years)
Mehra et al. 2011 [2]	30.6^b^	1–1–8	NA	NA	80% (2 years) 22.5% (5 years)	DSS 80% (2 years) DSS 22.5% (5 years) RFS 67% (2 years) RFS 8.3% (5 years)
Munir et al. 2012 [4]	24^a^ (22–26)	0–0–2	NA	0–3–5	NA	NA
Sargi et al. 2010 [22]^c^	28^a^ (3.7–79.8)	2–0–0	45.2^a^	2–0–0	Mean 72.9 months	Mean DFS 42.7 months
Wierzbicka et al. 2016 [23]	31.7^a^ 36^b^ (36–60)	0–4–3	12.7^a^ 8^b^	2–1–6	66.7% (3 years)^d^	DSS 77.8% (3 years)^d^ DFS 64.8% (3 years)^d^
Yokoyama et al. 2016 [11]	34.9^b^ (8–66)	NA	NA	NA	NA	NA

Abbreviations: D = distant recurrence; DFS = disease free survival; DOC = dead of other causes; DOD = dead of disease; DSS = disease specific survival; L = local recurrence; LRC = loco‐regional control; M = mastoidectomy; NA = not available; NED = no evidence of disease; OS = overall survival; *R* = regional recurrence; RFS = recurrence free survival.

^a^ Presented as mean.

^b^ Presented as median (range).

^c^ Raw data not extractable.

^d^ Calculated based on raw data.

## 4 Discussion

TPR consists of en bloc removal of a variable portion of the temporal bone combined with the parotid gland and adjacent structures depending on the extent of the tumor. This surgical concept was proposed by Allen in 1966 [14], who first described the procedure in two patients with a malignant parotid tumor extending to the temporal bone. The advantage of achieving free surgical margins with excellent exposure of the surgical field was emphasized. Since that time, due to the indications and complexity of the surgical technique, TPR received limited attention. In 1993, Leonetti et al. [21] revived the concept of combining lateral skull base procedures en bloc with radical parotid surgery to treat advanced parotid neoplasms, thus offering a more oncologically sound resection with superior local tumor control. In recent decades, TPR has been included in series together with more limited procedures reporting on malignant primary or secondary parotid tumors, accounting for 13%–28% of the total procedures performed [25, 26, 27]. According to the more recent literature [2, 4, 5, 6, 7, 11, 15, 16, 20, 21, 22, 23, 24], the need for extensive en bloc surgery combining lateral skull base approaches with parotidectomies and adjuvant therapies for aggressive parotid tumors prompted a reappraisal of en bloc temporal procedures [9]. To the best of our knowledge, this is the first systematic review reporting on the worldwide experience of TPRs for parotid malignancies.

### 4.1 Analysis of Prognostic Factors and Survival Outcomes

In this study, we aimed to analyze the state‐of‐the‐art of en bloc TPR for salivary gland tumors. Our systematic review identified 13 studies encompassing 336 patients, who were mostly diagnosed with a primary parotid malignancy (74.4%). As expected, a high heterogeneity in histopathological diagnoses was found, thus preventing a subgroup analysis of results and survival. Only 1 study [20] analyzed a series of 16 patients with adenoid cystic carcinoma of the parotid including the temporal bone. At 5‐year follow‐up, the authors reported three local and three distant recurrences, with four patients (25%) deceased for the disease. In the retrieved articles, TPR variously combined surgery on the parotid gland and temporal bone, with radical parotidectomy (65.7%) and lateral temporal bone resection (45.4%) being the most commonly performed procedures, respectively.

TPR achieved free margins in 74.5% of patients, while in the remaining 25.5% margins were positive or close. In the series by Gidley et al. [6], the rate of free‐margin resections when combining temporal bone surgery with parotidectomy in advanced‐stage parotid malignancies was 78.2%; the result was even higher in the study by Yokoyama et al. [11], with a 100% rate of negative margins. However, assessment of surgical margins still poses a challenge to both surgeons and pathologists, who need to closely collaborate in the orientation of the en bloc specimen. At present, margin assessment represents a crucial aspect that has an impact on adjuvant treatment selection and survival, which has received little attention in the literature [10]. Notably, Carlson et al. [7] highlighted the importance of achieving free margins in the proximal intratemporal facial nerve by combining parotidectomy with temporal bone resection. However, they stated that in the absence of radiologically evident temporal bone invasion, the need to perform TPR is controversial. Conversely, the same authors recommended TPR for patients with upper and lower facial nerve division paresis, or when the tumor was adjacent to or abutted the stylomastoid foramen. Similarly, Kadakia et al. [16] cautioned that patients with advanced parotid malignancy should be treated aggressively with parotidectomy and lateral temporal bone resection, regardless of bone involvement, due to the increased risk of local recurrence in those treated with a combination of parotidectomy and simple mastoidectomy. In 2021, Tanavde et al. [28], based on National Cancer Database data including 16,595 parotidectomies and 134 parotidectomies with temporal bone resections, identified the following factors at multivariable analysis as being associated with temporal bone resections in primary parotid malignancies: (i) advanced stage, (ii) high grade, and (iii) adenoid cystic carcinoma. In addition, patients treated at an academic center were more likely to undergo parotidectomy combined with temporal bone resections, thus emphasizing the advanced expertise required for such a treatment modality.

While the studies included in the present review lack homogeneity in reporting TPR outcomes and include patients with different histologies, the results suggest that en bloc TPR increases local control of the disease [2, 5, 6, 7, 15, 21, 28]. Overall, among patients with disease relapse at follow‐up, only 36% and 12.6% experienced local and regional recurrence, respectively. The en bloc resection demonstrated significantly better local disease control compared to parotidectomy combined with mastoidectomy. Kadakia et al. [16] reported a local recurrence rate of 3.3% in the en bloc resection group versus 21.6% in the parotidectomy plus mastoidectomy group (*p* = 0.002), reinforcing the oncologic advantage of a more extensive resection. In our systematic review, distant metastases were the most frequent cause of failure, with a rate of 51.4%. This finding was evident in the study by Aslier et al. [24] in which all four patients with disease relapse developed distant metastases. Similarly, in two other series [2, 15], distant metastases occurred in 83% and 40% of recurrences, respectively. Unfortunately, to the best of our knowledge, there are no comparative studies investigating OS as the main survival outcome in a series of TPR; thus, the efficacy of this surgical procedure on OS has yet to be determined.

This systematic review highlighted an overall paucity of data on survival outcomes, with only a single paper reporting OS, DSS, and RFS at 2 and 5 years [2]. In addition, outcomes were determined at different time points (2, 3, or 5 years) (Table 4). Unfortunately, local‐, regional‐, and distant‐recurrence free survivals were not calculated, and thus subgroup recurrence estimation could not be determined. Three‐year and 5‐year DSS ranged between 79%–66.7% and 72%–22.5%, respectively.

### 4.2 Strengths and Limitations

Our literature research was conducted with a systematic approach by defining strict inclusion and exclusion criteria, minimizing ambiguity in the selection process, and promoting critical evaluation of the subsequent conclusions. The present study has several limitations. (i) The observational retrospective design of all the studies included leads to intrinsic biases and can limit the generalizability of findings. (ii) The large heterogeneity of tumor features in each study (e.g., inclusion of various salivary gland histologies, presence of primary and secondary tumors) can influence clinical behavior and outcomes. (iii) Non‐standardized surgical techniques with different degrees of temporal bone resection, as well as variable adjuvant treatment modalities, reflect the lack of shared guidelines and the promising role of TPR in the management of parotid malignancies extending into or abutting the temporal bone. (iv) The lack of raw data in many papers prevented a pooled analysis of outcomes and, possibly, analyses of subgroups. (v) Missing data: many series suffered from incomplete reporting, especially considering survival outcomes and margin status. OS, DSS, and DFS were sparsely determined among studies, and the high variability in the duration of follow‐up further limited the possibility of assessing long‐term outcomes and recurrence rates. (vi) Patients were treated over several decades (1987–2016): the parallel evolution of surgical techniques may have improved outcomes, thus affecting the homogeneity of survival data.

Overall, despite the above‐mentioned limitations, this study provided insights into the role of TPR in the management of malignant tumors of the parotid gland with temporal bone involvement. Prospective studies with homogeneity of histologies, standardized treatment protocols, well‐defined survival endpoints, and adequate follow‐up are advocated to define the precise role of the en bloc technique in this field.

## 5 Conclusions

Surgical management of parotid gland malignancies represents a complex challenge, especially in cases where extraglandular extension to surrounding tissues such as the ear and temporal bone are present. As aptly expressed by Gidley in “The oncology of otology” [29], providing comprehensive care for patients with head and neck cancer demands multi‐faceted expertise. In this setting, a detailed knowledge of temporal bone anatomy and skills in skull base surgery, together with a sound knowledge of surgical oncologic principles, represent a step forward in the management of patients with parotid gland tumors involving the temporal bone. This underscores the importance of collaboration and innovation among dedicated surgeons in this field to meet the needs of patients with complex head and neck malignancies.

This systematic review highlights that the surgical approach with en bloc TPR, already described in the past, seems to be a viable strategy to improve local control of the disease in parotid gland malignant tumors abutting the temporal bone. This approach, which in most patients is the first step of multidisciplinary treatment, could improve the quality of resection by providing a high rate of negative margins.

Given the heterogeneity in histopathology of parotid malignancies, oncologic features, margin status, and adjuvant therapies, the findings of this review should be interpreted cautiously. While TPR appears to improve local control and margin status, further standardized, single‐histology, and prospective studies are needed to validate these conclusions. The absence of direct comparisons with other treatment modalities and heterogeneity among cases necessitate further research to substantiate its efficacy.

## Conflicts of Interest

The authors declare no conflicts of interest.

## Supporting information


**Supporting Information A.** Search queries.
**Table S1.** Quality assessment of the included studies.
**Figure S1.** Risk of bias assessment for non‐randomized studies (ROBIN‐I tool).
