# Supplementary material for: Temporal‐Parotid Resection for Malignant Parotid Tumors: A Systematic Review
Source: Laryngoscope. 2025 May 14;135(11):4009–19. doi: 10.1002/lary.32267 (PMC12645363; doi:10.1002/lary.32267)
Supplement: Supplementary file 1 — Supporting Information A. Search queries. Table S1. Quality assessment of the included studies. Figure S1. Risk of bias assessment for non‐randomized studies (ROBIN‐I tool). [file LARY-135-4009-s001.docx]

**SUPPLEMENT A**

**SEARCH QUERIES**

**PUBMED**

("Salivary gland"[All Fields] OR "Parotid gland"[All Fields] OR ("Parotid gland"[MeSH Terms] OR ("parotid"[All Fields] AND "gland"[All Fields]) OR "Parotid gland"[All Fields] OR "parotid"[All Fields] OR "parotids"[All Fields] OR "parotide"[All Fields] OR "parotideal"[All Fields])) AND ("cancer s"[All Fields] OR "cancerated"[All Fields] OR "canceration"[All Fields] OR "cancerization"[All Fields] OR "cancerized"[All Fields] OR "cancerous"[All Fields] OR "neoplasms"[MeSH Terms] OR "neoplasms"[All Fields] OR "cancer"[All Fields] OR "cancers"[All Fields] OR ("carcinoma"[MeSH Terms] OR "carcinoma"[All Fields] OR "carcinomas"[All Fields] OR "carcinoma s"[All Fields]) OR ("neoplasm s"[All Fields] OR "neoplasms"[MeSH Terms] OR "neoplasms"[All Fields] OR "neoplasm"[All Fields]) OR "Advanced stage"[All Fields] OR ("malign"[All Fields] OR "malignance"[All Fields] OR "malignances"[All Fields] OR "malignant"[All Fields] OR "malignants"[All Fields] OR "malignities"[All Fields] OR "malignity"[All Fields] OR "malignization"[All Fields] OR "malignized"[All Fields] OR "maligns"[All Fields] OR "neoplasms"[MeSH Terms] OR "neoplasms"[All Fields] OR "malignancies"[All Fields] OR "malignancy"[All Fields]) OR "Malignant tumor"[All Fields]) AND ("Extended parotidectomy"[All Fields] OR ("mastoid"[MeSH Terms] OR "mastoid"[All Fields] OR "mastoids"[All Fields] OR "mastoidal"[All Fields] OR "mastoiditis"[MeSH Terms] OR "mastoiditis"[All Fields]) OR ("mastoidectomy"[MeSH Terms] OR "mastoidectomy"[All Fields] OR "mastoidectomies"[All Fields]) OR "Temporal bone"[All Fields] OR "En bloc resection"[All Fields])

Filters: NO

**SCOPUS**

("Salivary gland" AND "Parotid gland" AND Parotid) AND (Cancer OR Carcinoma OR Neoplasms OR "Advanced stage" OR Malignancy OR "Malignant tumor") AND ("Extended parotidectomy" OR

Mastoid OR Mastoidectomy OR "Temporal bone" OR "En bloc resection")

Filters: NO

**EMBASE**

1 "Salivary gland".mp. or salivary gland/ 53651

2 "Parotid gland".mp. 29812

3 Parotid.mp. 36019

4 Cancer.mp. or malignant neoplasm/ 4471782

5 Carcinoma.mp. or carcinoma/ 1370942

6 Neoplasms.mp. or neoplasm/ 695086

7 "Advanced stage".mp. 54203

8 Malignancy.mp. 320081

9 "Malignant tumor".mp. or malignant neoplasm/ 127126

10 "Extended parotidectomy".mp. 27

11 Mastoid.mp. or mastoid/ 10798

12 Mastoidectomy.mp. or mastoidectomy/ 6665

13 "Temporal bone".mp. or temporal bone/ 20249

14 "En bloc resection".mp. 10321

15 1 or 2 or 3 78950

16 4 or 5 or 6 or 7 or 8 or 9 5113230

17 10 or 11 or 12 or 13 or 14 42408

18 15 and 16 and 17 483

**Table S1:** Quality assessment of the included studies.

| Author | Quality^18^ |
| --- | --- |
| Amin et al. 2017^5^ | Fair |
| Aslier et al. 2019^24^ | Fair |
| Carlson et al. 2015^7^ | Fair |
| Gidley et al. 2011^6^ | Good |
| Kadakia et al. 2017^16^ | Poor |
| Leonetti et al. 1993^21^ | Good |
| Leonetti et al. 2008^20^ | Fair |
| Martin et al. 2017^15^ | Fair |
| Mehra et al. 2011^2^ | Good |
| Munir et al. 2012^4^ | Poor |
| Sargi et al. 2010^22^ | Good |
| Wierzbicka et al. 2016^23^ | Fair |
| Yokoyama et al. 2016^11^ | Poor |

**Figure S1.** Risk of bias assessment for non-randomized studies (ROBIN-I tool).

**
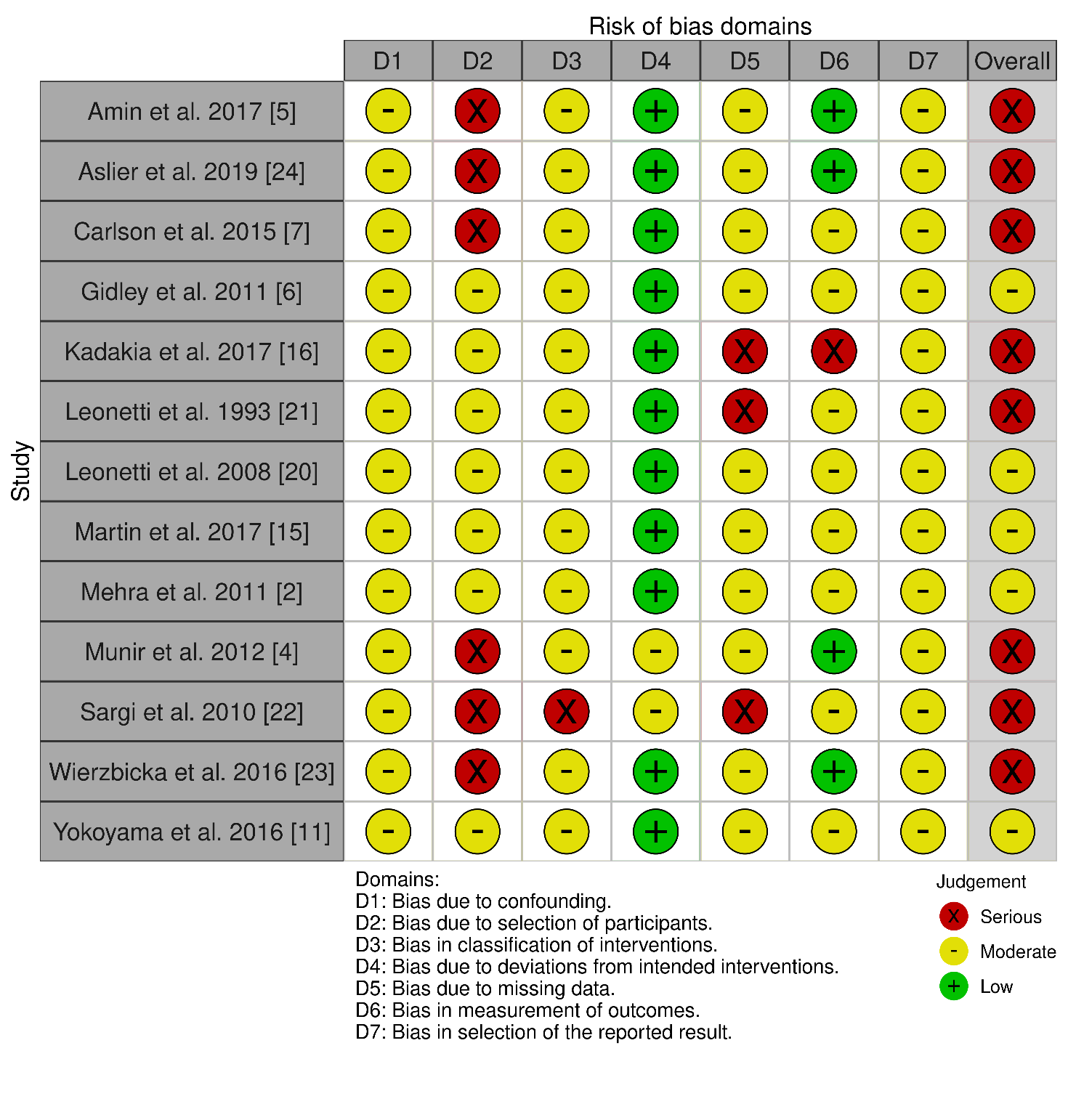
**
